# Integrating primary care and social services for older adults with multimorbidity: policy implications

**DOI:** 10.3399/BJGPO.2021.0035

**Published:** 2021-06-09

**Authors:** Sam Hodgson, Glenn Simpson, Paul Roderick, Hazel Everitt, Paul Little, Miriam Santer, Hajira Dambha-Miller

**Affiliations:** 1 Primary Care Research Centre, University of Southampton, Southampton, UK; 2 Department of Population Health, University of Southampton, Southampton, UK

**Keywords:** Integrated care, older adults, multimorbidity, primary care, social services, general practice

## Introduction

Over the next 20 years, the proportion of over-65s in the UK will rise by a quarter,^1^ around two-thirds of whom will live with multimorbidity (multiple long-term conditions).^2^ This change in demographic is likely to lead to a significant growth in care needs,^3^ further increasing demand on primary care and social services. In response, policymakers have been trying to accelerate the drive towards integrated care^4^ to deliver service efficiencies, cost savings and, concomitantly, improvements in outcomes for patients and service users. To this end, a variety of integrated care pilots have been trialled.^4^ There have been concerns that some of these testbeds have been rolled out nationally without a strong evidence base or comprehensive evaluation, for example, ’social prescribing’ initiatives.^5^ This has led to uncertainty around the efficacy of integrated care initiatives, and no consensus on how best to integrate primary care and social services for older adults with multimorbidity.

To address this gap in the evidence base, we carried out a mixed methods programme of research. This included a scoping review of the literature,^6^ and a qualitative interview study to elicit key stakeholder views on drivers and barriers to integration of primary care and social services in England. Stakeholders included patients, care service users, carers, primary and secondary care clinicians, social prescribers, community nurses, social workers, voluntary sector workers, and multiple other relevant individuals.^7^ We used Valentijn’s Rainbow Model of Integrated Care^8^ as an analytical and spatial lens to interrogate and understand both the literature we identified and the empirical data derived from the semi-structured interviews. This conceptual framework describes integration occurring at and across a range of scales: the whole system level (macro-level integration); the organisational and professional level (meso-level); and the level of clinical and service integration (micro-level).

In this article, we summarise our key findings and propose policy learning points.

### Key findings and recommendations

It is increasingly recognised that, to be effective, comprehensive health and social care integration must occur across multiple scales, joining up care both vertically (from the micro- to macro-level) and horizontally among the range of service providers.^9,10^ In practice, our work showed that efforts to drive forward integration are mainly focused on two scales: first, micro-scale clinical initiatives aimed to integrate care at the point of delivery, for example by having a social worker employed in a local emergency department; second, to a lesser extent, integration is also focused on the meso-level, in the form of interprofessional collaboration and joint working arrangements between organisations. An example of integration at this scale is roles jointly funded by both health and social care. While it has long been postulated that a whole-system approach is necessary to address the needs of older adults with multimorbidity,^10^ there is limited research evidence of multi-level vertical and horizontal integration across service providers in an English context.^9^ Currently, health and social care in England is delivered by separate organisations with striking differences in funding, political oversight, and delivery models; for example, health care is free at the point of delivery, but social care is not. In this context, we argue that there is a need to increase macro-scale integration at higher organisational and strategic levels, involving multiple providers, including the private and voluntary sectors.

When attempting to integrate health and social care, a critical factor that is often overlooked is the lack of time given to integrated care programmes to establish an effect. Although there have been calls for integrated care programmes to be given time to mature,^11^ implementation has repeatedly taken place over relatively short-term periods.^12^ For example, ‘Vanguard’ sites were established in England in 2015, quickly followed by interventions such as ‘social prescribing’, ‘care navigation’, and then the Integrated Care Systems programme.^12^ Significantly, none of these underwent thorough evaluation prior to national rollout.^5,13^ Furthermore, integration has been a challenge in the context of a repeated ’churn’ of new policy programmes, leadership figures, organisational structures, and funding streams. This highlights the challenge faced by policymakers between encouraging innovation while allowing time for new models of integrated care to establish, mature, and potentially reach operational efficiency prior to evaluation. There is a need to work with policy communities to use available evidence as best as possible to achieve this.

Stakeholder experiences and published literature consistently highlight enablers of integrated care which could be harnessed by policymakers.^4,11,14^ First, although the pivotal role of dynamic leaders in driving and sustaining integrated working is repeatedly emphasised in earlier research,^9^ policymakers to date have given limited attention to this enabler of integrated care. Embedding leadership systematically presents challenges, in particular sustainability of change when dynamic leaders become less dynamic or leave their role, but one way of achieving this could be through wider rollout of local ’champion‘ roles and leadership training programmes. Second, the development of ’link‘ or ‘interface‘ roles — such as ’Care Navigators’ — who are able to provide integration between existing services potentially limits the need for wider service re-design at the system or organisational levels by enabling integration within existing models of care.^14^ Their location at potential ‘pinch points’ in care systems enables care transitions across sectoral boundaries and offers patients, care service users, and carers support in navigating complex care systems. Third, co-location and shared working spaces can facilitate interprofessional relationship-building and trust, which are essential to establishing sustainable, integrated working arrangements and multidisciplinary teams. Harnessing the potential of these enablers within whole-system integration might allow policymakers to overcome historic tensions between central policy and local service delivery agendas, and in doing so facilitate development of better models of integrated care.

## Conclusion

We identified common themes in the published literature and stakeholder interviews highlighting drivers of, and barriers to, integration between primary care and social services for older adults with multiple long-term health conditions. In the context of an increasingly ageing population with growing care needs,^3^ learning from past integration experiences is essential to addressing future challenges. A critical issue for systems of integrated care is to deliver holistic, whole-system structures, which require high-level policy initiatives while allowing flexibility for local solutions. This can only be achieved with sufficient political will and urgent action.
